# Complete genome sequence of a metallo-β-lactamase-producing *Aeromonas dhakensis* strain, RYU-Ah62, isolated from a patient with an abdominal abscess

**DOI:** 10.1128/mra.00010-24

**Published:** 2024-06-25

**Authors:** Naoya Nishiyama, Kohei Uechi, Wakako Arakaki, Daisuke Utsumi, Yukuto Sato, Masashi Nakamatsu, Takeshi Kinjo, Kazuko Yamamoto

**Affiliations:** 1 First Department of Internal Medicine, Division of Infectious, Respiratory, and Digestive Medicine, University of the Ryukyus Graduate School of Medicine, Okinawa, Japan; 2 Division of Clinical Laboratory and Blood Transfusion, University of the Ryukyus Hospital, Okinawa, Japan; 3 Department of Dermatology, Advanced Medical Research Center, Graduate School of Medicine, University of the Ryukyus, Okinawa, Japan; 4 Research Laboratory Center, Faculty of Medicine, University of the Ryukyus, Okinawa, Japan; Loyola University Chicago, Chicago, Illinois, USA

**Keywords:** *Aeromonas*, metallo-β-lactamase

## Abstract

*Aeromonas dhakensis* is highly virulent but often misidentified in clinical settings. The entire genome sequence of a metallo-β-lactamase-producing *A. dhakensis* strain from a clinical specimen has been presented in this study. The genome comprised a single chromosome of 4.89 Mbp with 61.6% G + C content.

## ANNOUNCEMENT

*Aeromonas dhakensis* is frequently misidentified in clinical settings as *Aeromonas hydrophila* (1). Bacteremia is more lethal when caused by *A. dhakensis* than by other *Aeromonas* species (2). *Aeromonas dhakensis*, like A. *hydrophila*, produces CphA, a metallo-β-lactamase, and is carbapenem-resistant (3). This study presents the whole genome sequence of a clinical *A. dhakensis* isolate obtained from a 40-year-old patient with an abdominal abscess after recurrent colon cancer surgery at the University of the Ryukyus Hospital. Fluid from the abscess was cultured overnight on MacConkey agar at 37°C. The isolate was identified as *A. hydrophila* using VITEK MS (bioMerieux, Craponne, France) and the Voges-Proskauer test. Antimicrobial susceptibility testing was conducted using VITEK II (bioMerieux). The isolate displayed a high meropenem minimal inhibitory concentration (4 µg/mL) and was suspected to have *cphA* based on PCR results (4). However, the amplified product was approximately 200 bp longer than *cphA* typical length.

The isolate was subcultured on 5% sheep blood agar (SBA) (Becton Dickinson, Franklin Lakes, NJ, USA) and incubated overnight at 37°C. Genomic DNA was extracted from colonies in SBA using the magLEAD 12gC system (Precision System Science, Chiba, Japan). Sequencing libraries were prepared using Rapid Barcoding Sequencing (Oxford Nanopore Technologies, Oxford, UK) and Nextera XT DNA Sample Preparation Kit (Illumina, San Diego, CA, USA). Long-read sequencing was conducted on the MinION platform (Oxford Nanopore Technologies), equipped with the R9.4 flow cell. Base calling was performed using Guppy v6.4.8 (Oxford Nanopore Technologies), yielding 97,498 reads (mean read length, 8,960.3 bp; N50 read length, 13,925 bp) and a total sequence length of 873 Mbp, with approximately 170 × coverage. Reads were filtered using NanoFilt v2.8.0 (5), and only those with a length of ≥1,000 bp were retained. The first 50 bp of each read was trimmed. The genome was assembled using Flye v2.9.2 (6) and polished using Medaka v1.7.3 (Oxford Nanopore Technologies). Short-read sequencing was conducted using MiSeq (Illumina), yielding 150 bp paired-end reads. The number of raw reads for short-read pairs was 255,378. Reads were trimmed using Fastp v.0.6.5 (7). The long-read assembly was polished once using short reads with Pilon v1.24 (8). Assembly completeness was assessed using CheckM v1.1.6 (9). Default settings were applied. Annotation was performed using DFAST v1.1.0 from DDBJ (10). The average nucleotide identity (ANI) value was calculated using FastANI (11) in DFAST. Finally, antimicrobial resistance gene content was determined using ResFinder v4.1 (12–14). The assembled genome consisted of a 4.89 Mbp single chromosome with a 61.6% G + C content and 161 × genome coverage. The isolate was identified as *A. dhakensis* based on a 97.2% ANI value for the type strain (strain CIP 107500; GenBank accession number GCF_000820305.1) and 99.8% completeness. ResFinder v4.1 (12–14) analysis detected two resistance genes, *cphA1* (identity, 94.7%) and *ampH* (identity, 95.8%). BLAST (15, 16) analysis revealed a complete match between *cphA1* and *A. dhakensis* DNA sequences, including a CphA-type class B metallo-β-lactamase in strain A2-155 (AB765405). The sequence had an insertion of 218 bases in *cphA*. Overall, 4,391 genes were predicted in this strain.

The University of the Ryukyus Ethics Committee waived the need for informed consent as the study involved no human subjects.

## Data Availability

Sequencing data have been deposited in DDBJ under accession no. AP028321 and the Sequence Read Archive (SRA) under accession nos. DRR518146 and DRR518147.
